# LiNi_0.4_Co_0.3_Mn_0.3_O_2 _thin film electrode by aerosol deposition

**DOI:** 10.1186/1556-276X-7-64

**Published:** 2012-01-05

**Authors:** Icpyo Kim, Tae-Hyun Nam, Ki-Won Kim, Jou-Hyeon Ahn, Dong-Soo Park, Cheolwoo Ahn, Byong Sun Chun, Guoxiu Wang, Hyo-Jun Ahn

**Affiliations:** 1 School of Materials Science and Engineering, ERI, Gyeongsang National University, Jinju, 660-701, South Korea; 2 Department of Chemical and Biological Engineering, Gyeongsang National University, Jinju, 660-701, South Korea; 3 Functional Materials Division, Korea Institute of Materials Science, Changwon, 641-831, South Korea; 4 Centre for Clean Energy Technology, Department of Chemistry and Forensic Science, University of Technology Sydney, Broadway, Sydney, NSW 2007, Australia; 5 ReSEAT Program, KISTI, Daejeon, 305-806, South Korea

**Keywords:** thin film, aerosol deposition, battery

## Abstract

LiNi_0.4_Co_0.3_Mn_0.3_O_2 _thin film electrodes are fabricated from LiNi_0.4_Co_0.3_Mn_0.3_O_2 _raw powder at room temperature without pretreatments using aerosol deposition that is much faster and easier than conventional methods such as vaporization, pulsed laser deposition, and sputtering. The LiNi_0.4_Co_0.3_Mn_0.3_O_2 _thin film is composed of fine grains maintaining the crystal structure of the LiNi_0.4_Co_0.3_Mn_0.3_O_2 _raw powder. In the cyclic voltammogram, the LiNi_0.4_Co_0.3_Mn_0.3_O_2 _thin film electrode shows a 3.9-V anodic peak and a 3.6-V cathodic peak. The initial discharge capacity is 44.6 μAh/cm^2^, and reversible behavior is observed in charge-discharge profiles. Based on the results, the aerosol deposition method is believed to be a potential candidate for the fabrication of thin film electrodes.

## Introduction

Batteries can be applied to microelectronic and portable devices as power sources [1-3]. Also, many endeavors have been made to develop batteries for high power and energy for electric vehicles [4,5]. Although lithium-ion batteries, among all other batteries, are the most promising type owing to their large energy storage density, commercial lithium-ion batteries contain a flammable liquid electrolyte, which has induced safety concerns. In order to mitigate the safety issue, an all-solid-state battery is a viable candidate as it is composed of thin film electrodes and a solid electrolyte. Moreover, the thin film electrode usually is composed of an active material without a binder. Owing to these advantages, many studies have been conducted to fabricate all-solid-state batteries through various methods, such as pulsed laser deposition [6-13], electrostatic spray deposition [14-16], and sputtering deposition [17-26]. Although these methods are very efficient for the preparation of thin film electrodes, they have several disadvantages, such as their complex fabrication processes, difficulty in controlling the composition of the thin film, and their low deposition rate.

Aerosol deposition method was recently developed that differs from aerosol flame deposition in which the materials are prepared through a hydrolysis reaction of aerosol precursor solutions by flame [27]. The aerosol deposition method can be used for various applications, such as biomaterial and ceramic sensors [28-30]. In the aerosol deposition method, powder is mixed with gas to make an aerosol, and this aerosol is ejected onto the substrate to form a thin film. In other words, the aerosol deposition is a room-temperature impact-consolidation method. Thus, the aerosol deposition method has excellent advantages. These include its room temperature process, high deposition rate, high adhesion strength, easy control of the composition of the thin film, and its simple process. Furthermore, the aerosol deposition method does not require high vacuum devices, and the bare powder can be used directly without a pretreatment.

LiNi_0.4_Co_0.3_Mn_0.3_O_2 _in the LiNi_x_Co_y_Mn_z_O_2 _system was chosen as an active material on the account of its low cost, low toxicity, thermal stability, high capacity, and good cycle life [31,32]. Xie et al. [25] recently reported a LiNi_0.33_Mn_0.33_Co_0.33_O_2 _thin film electrode prepared via a sputtering method. The LiNi_0.33_Mn_0.33_Co_0.33_O_2 _thin film electrode presented excellent results such as a high discharge capacity of more than 120 mAh/g. However, there was no report on the LiNi_0.4_Co_0.3_Mn_0.3_O_2 _thin film electrode. A complex conventional procedure was undertaken to deposit this thin film in their study. The aerosol deposition method was believed to have the ability to simplify this complex procedure, and no report has been made on using this method for the preparation of the thin film electrode.

In this study, a LiNi_0.4_Co_0.3_Mn_0.3_O_2 _thin film was prepared by aerosol deposition, and its electrochemical property was characterized. From these results, the feasibility of aerosol deposition as a new preparation method for thin film electrodes was discussed.

### Experimental details

**Figure 1:**
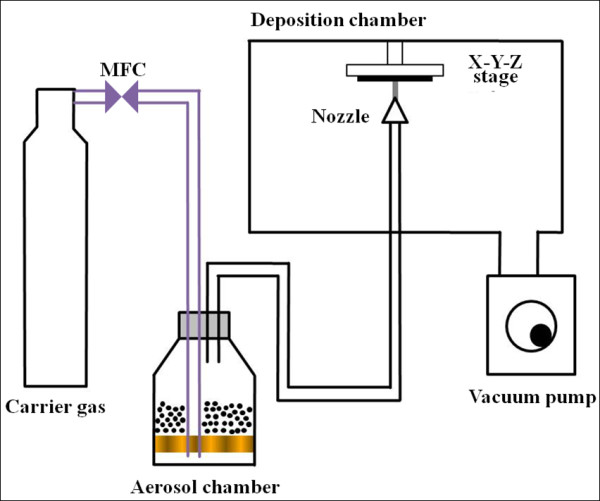
**Schematic diagram of aerosol deposition**.

We prepared LiNi_0.4_Co_0.3_Mn_0.3_O_2 _thin film electrodes from the LiNi_0.4_Co_0.3_Mn_0.3_O_2 _raw powder, which was purchased from DAEJUNG EM in Buchun-City, Korea and was used without any special pretreatment using the aerosol deposition apparatus (built in-house) as shown in Figure 1. Stainless steel (SUS304) was used as a substrate. The detailed AD procedure was described in our previous report [33].

To investigate the crystal structures, the LiNi_0.4_Co_0.3_Mn_0.3_O_2 _powder and thin film electrodes were analyzed by an X-ray diffractometer (D8 Bruker; Karlsruhe, Germany) employing Cu Kα radiation. A field emission scanning electron microscope [FESEM] (Philips XL30S FEG; Philips, Amsterdam, Netherlands) was used for clarifying the surface morphologies. For the measurement of electrochemical properties, a Swagelok-type cell was employed. The thin film electrodes were used as working electrodes, and a lithium metallic foil was designated as counter electrode. The electrolyte solution was 1 mol LiPF_6 _in EC + DEC (1:1 (*v*/*v*)). The assemblies of the cells were conducted in an Ar-filled glove box. Potentiostatic tests were carried out at a sweep rate of 0.1 mV/s between 2.5 and 4.2 V for the thin film electrode, and galvanostatic tests were performed at a constant current density of 1 μA/cm^2 ^in the same voltage range.

## Results and discussions

**Figure 2:**
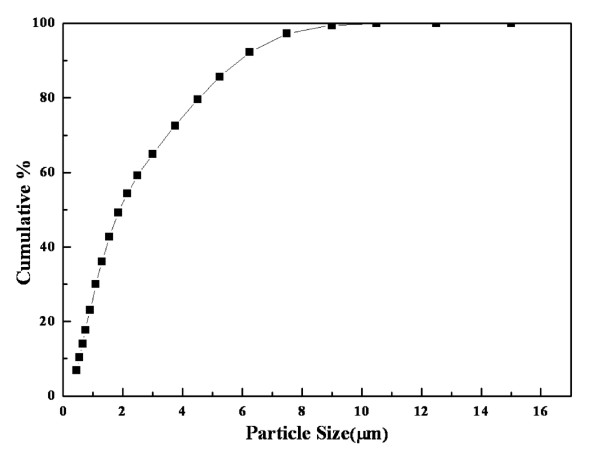
**The cumulative distribution of particle size of LiNi_0.4_Co_0.3_Mn_0.3_O_2 _raw powder**.

**Figure 3:**
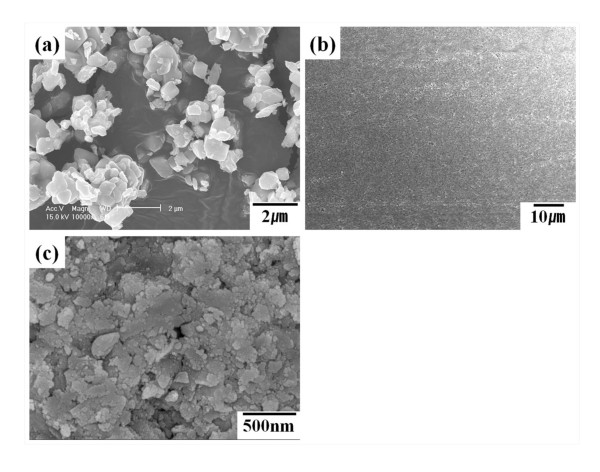
**SEM micrographs**. LiNi_0.4_Co_0.3_Mn_0.3_O_2 _(**a**) raw powder and thin film electrode at a magnification of (**b**) ×1,000 and (**c**) ×40,000.

In the aerosol deposition method, particle size of the starting powder was an important experimental factor, which was measured by WINDOX 5 (HELOS Particle Size Analysis; Sympatec Inc., Lawrenceville, NJ, USA). Figure 2 presents the cumulative distribution of the particle size of LiNi_0.4_Co_0.3_Mn_0.3_O_2 _raw powder, which ranged from the submicron to 11 μm. The average particle size was 1.9 μm. Figure 3 shows FESEM images of the LiNi_0.4_Co_0.3_Mn_0.3_O_2 _raw powder and thin film electrode. The LiNi_0.4_Co_0.3_Mn_0.3_O_2 _powder presented an agglomeration of small particles. This LiNi_0.4_Co_0.3_Mn_0.3_O_2 _powder was deposited uniformly, and the thin film had a rough and flat surface in low magnification. In high magnification, the thin film electrode consisted of fine particles of less than several hundred nanometers. During the aerosol deposition process, the original particles could be crushed into fine particles upon the moment of impact on the substrate. These fractured fine particles strongly attached to the substrate, as explained in a previous report [34]. Thus, based on the particle size analysis result, the original particles that were considered became small by more than half of the original size. The thickness of the thin film was about 2.6 μm as measured by α-step measurements, and 1 min was consumed for the deposition. Thus, the deposition rate of the thin film could be about 2.6 μm/min, which was much faster than that of conventional deposition methods.

**Figure 4:**
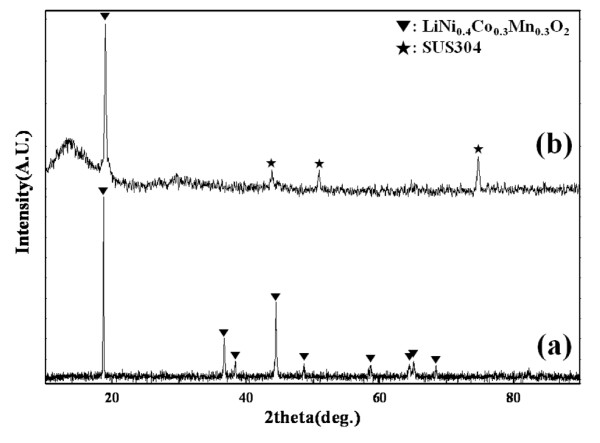
**XRD patterns of the (a) LiNi_0.4_Co_0.3_Mn_0.3_O_2 _raw powder and (b) thin film electrode**.

Because aerosol deposition is a shock-loading deposition method, it can induce severe strain or a change in the crystal structure of the thin film. In particular, it is well known that a LiNi_0.33_Co_0.33_Mn_0.33_O_2_-based material has a layered structure of α-NaFeO_2 _(*R-*3*m*) and that lithium ions lithiate/delithiate between these layers [32]. Thus, the crystal structure of the thin film can strongly affect its electrochemical properties. To investigate changes in the crystal structure of the LiNi_0.4_Co_0.3_Mn_0.3_O_2 _thin film, X-ray diffraction [XRD] measurements were conducted. Figure 4 shows the XRD patterns of the LiNi_0.4_Co_0.3_Mn_0.3_O_2 _raw powder and thin film electrode. The XRD patterns of the LiNi_0.4_Co_0.3_Mn_0.3_O_2 _raw powder confirmed the α-NaFeO_2 _(*R-*3*m*) structure, replicating the findings of a previous report [32,35]. However the XRD patterns of the thin film showed only one visible peak for LiNi_0.4_Co_0.3_Mn_0.3_O_2 _at 18° with three other peaks corresponding to the stainless steel substrate. This phenomenon has been reported for various thin films, and the preferred orientation of the thin film was suggested as an origin [9,25,36]. The same reason might be applied to our X-ray diffraction result. Moreover, the peak of the thin film was slightly broader than that of the raw powder, which may originate from the strain of the crystal structure or the small particle size as shown in Figure 3c.

**Figure 5:**
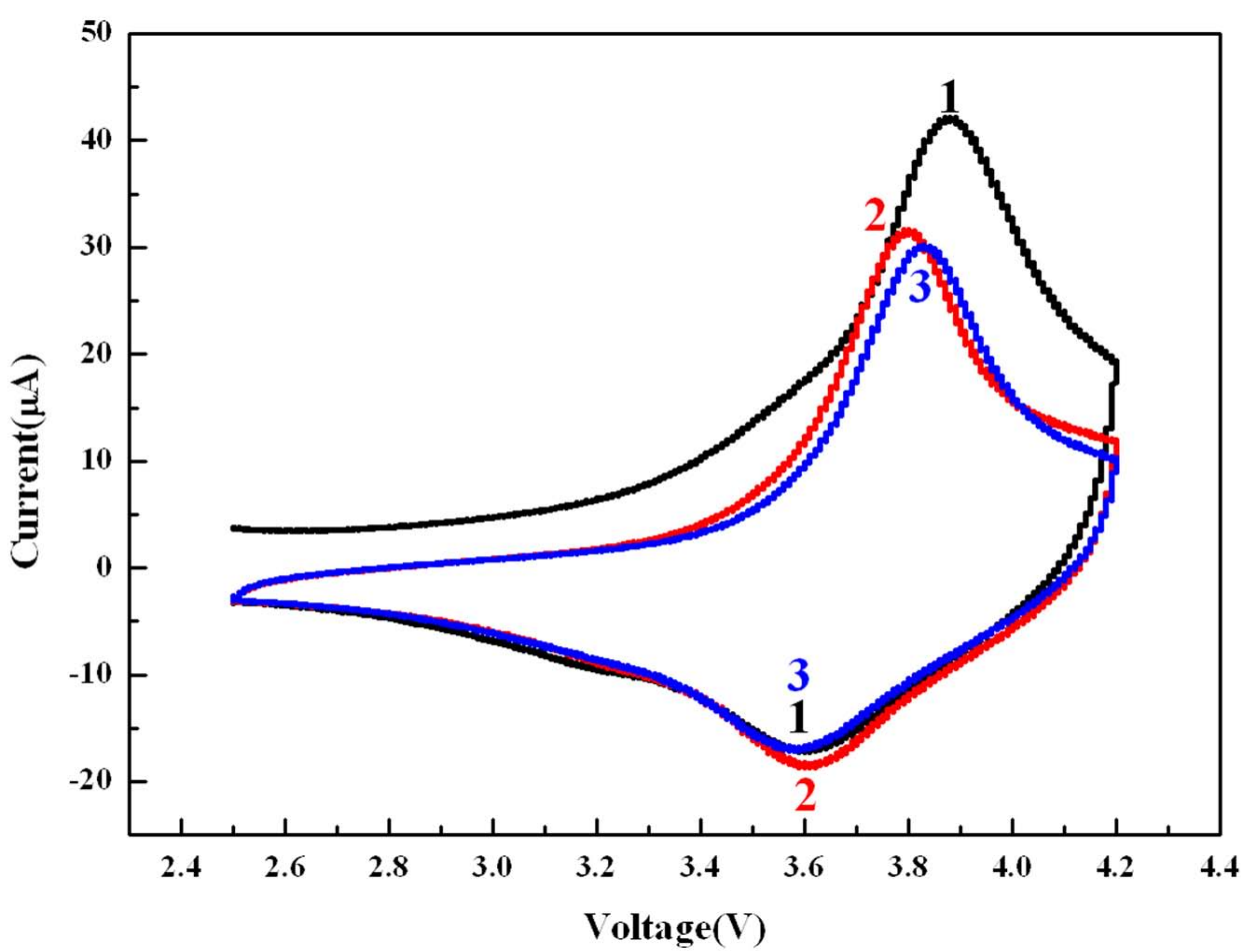
**Cyclic voltammogram of the LiNi_0.4_Co_0.3_Mn_0.3_O_2 _thin film electrode at a scan rate of 0.1 mV/s**.

Figure 5 introduces the cyclic voltammogram [CV] of the thin film electrode. The LiNi_0.4_Co_0.3_Mn_0.3_O_2 _thin film electrode showed a 3.88-V oxidation peak and a 3.6-V reduction peak in the first cycle. Since there has been no previous study on CV of the LiNi_0.4_Co_0.3_Mn_0.3_O_2 _thin film, previous results on LiNi_1/3_Co_1/3_Mn_1/3_O_2 _bulk electrodes by Shinova et al. and He et al. [37,38] were taken into account, and from comparison, a similarity of redox peak voltages was observed. The thin film electrode is believed to have electrochemical properties corresponding to those of the LiNi_0.4_Co_0.3_Mn_0.3_O_2 _bulk electrode, coinciding with the XRD result in Figure 4. In the second cycle, the reduction peak shifted slightly, but the oxidation peak appeared at 3.80 V and moved to a high voltage in the third cycle. This demonstrates that the rechargeable LiNi_0.4_Co_0.3_Mn_0.3_O_2 _thin film electrode can be fabricated for rechargeable all-solid-state batteries by aerosol deposition method. However, the redox peaks were broad, and the peak voltages shifted. The aerosol deposition method is based on the impact adhesion of particles, which means that the particles yield a large strain in itself from the impact. Thus, the broadness and the voltage shifts of redox peaks are believed to be attributed to the severe strain of particles.

**Figure 6:**
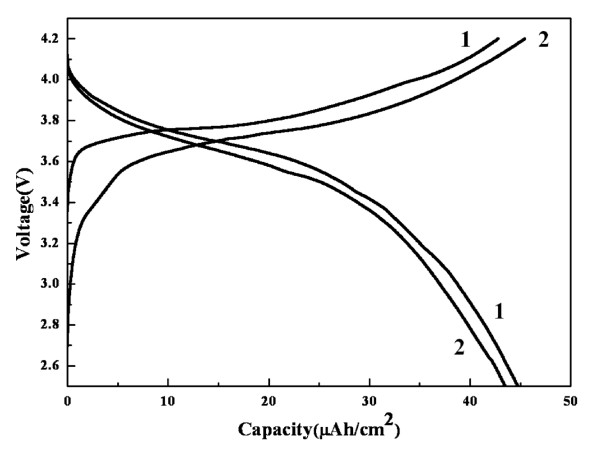
**The charge and discharge curves of the LiNi_0.4_Co_0.3_Mn_0.3_O_2 _thin film electrode**.

The charge-discharge curves of the LiNi_0.4_Co_0.3_Mn_0.3_O_2 _thin film electrode are presented in Figure 6. The thin film electrode yielded the first charge and discharge capacities, 42.8 and 44.7 μAh/cm^2^, respectively. In the second cycle, the charge capacity increased to 45.4 μAh/cm^2^, and the discharge capacity decreased to 43.5 μAh/cm^2^. Rechargeability of the thin film electrode was introduced in accordance with the CV result. In the previous report on amorphous Li[Ni_1/3_Co_1/3_Mn_1/3_]O_2 _positive electrode by Xie et al. [25], an irreversible capacity was presented at the first cycle, but the LiNi_0.4_Co_0.3_Mn_0.3_O_2 _thin film electrode exhibited this at the second cycle. The plateau voltages of the charge and discharge curves decreased in the second cycle. As described above, aerosol deposition is based on shock-loading solidification. Therefore, a large strain can be introduced into the thin film, which is released during initial cycles and induces the partial collapse or change of the crystal structure of the thin film; thus, the capacity and potential can be affected. The sloped flat region of the discharge curves could be attributed to several factors such as current density and crystal structure of the active material, but the current density of 1 μA/cm^2 ^was quite low compared to the capacity of 44.7 μAh/cm^2^. Thus, we believe that the damaged crystal structure also contributed the discharge behavior of the thin film electrode.

## Conclusions

The feasibility of the aerosol deposition method for the fabrication of thin film electrodes was investigated. LiNi_0.4_Co_0.3_Mn_0.3_O_2 _thin film electrode was prepared within 10 min and had a flat surface composed of fine particle with the α-NaFeO_2 _crystal structure. According to cyclic voltammogram measurement, the thin film electrode showed a 3.9-V anodic peak and a 3.6-V cathodic peak. The discharge capacity was 44.7 μAh/cm^2 ^with a 3.6-V plateau region. Based on these results, the aerosol deposition method is a good candidate for the fabrication of thin film electrodes, which can be used in all-solid-state rechargeable batteries.

## Competing interests

The authors declare that they have no competing interests.

## Authors' contributions

IK carried out the electrochemical experiments and drafted the manuscript. THN participated in the crystallographic studies, and KWK and JHA did the electrochemical studies. DSP and CA carried out the deposition of the thin film. BSC participated by proofreading the manuscript. GW participated in the analysis of the materials. HJA conceived the study and participated in its design and coordination. All authors read and approved the final manuscript.
